# Advances in the treatment of pancreatic cancer with traditional Chinese medicine

**DOI:** 10.3389/fphar.2023.1089245

**Published:** 2023-08-07

**Authors:** Yanhua Zhang, Hui Xu, Yue Li, Yang Sun, Xiaochun Peng

**Affiliations:** ^1^ Department of Physiology, School of Basic Medicine, Health Science Center, Yangtze University, Jingzhou, Hubei, China; ^2^ Department of Pathophysiology, School of Basic Medicine, Health Science Center, Yangtze University, Jingzhou, Hubei, China; ^3^ Department of Internal Medicine, Southern Medical University, Guangzhou, China

**Keywords:** pancreatic cancer, traditional Chinese medicine, apoptosis, metastasis, therapy

## Abstract

Pancreatic cancer is a common malignancy of the digestive system. With a high degree of malignancy and poor prognosis, it is called the “king of cancers.” Currently, Western medicine treats pancreatic cancer mainly by surgical resection, radiotherapy, and chemotherapy. However, the curative effect is not satisfactory. The application of Traditional Chinese Medicine (TCM) in the treatment of pancreatic cancer has many advantages and is becoming an important facet of comprehensive clinical treatment. In this paper, we review current therapeutic approaches for pancreatic cancer. We also review the protective effects shown by TCM in different models and discuss the potential molecular mechanisms of these.

## 1 Introduction

Pancreatic cancer is a common malignancy of the digestive system. About 90% of pancreatic cancers are adenocarcinomas. This cancer occurs mainly in older men, aged between 40 and 85 years. Both the incidence rate and mortality of pancreatic cancer are increasing and it estimated that by 2030, it will become the second leading cause of cancer-related deaths (Dhasmana et al., 2023). The cancer always starts insidiously; as a result, patients are often in advanced stages when they are diagnosed, leading to poor 5-year survival rates of 2–9% (McGuigan et al., 2018). In China, the incidence of pancreatic cancer has increased approximately six-fold in the past 20 years (Shen and Liu, 2009). The development of pancreatic cancer is associated with many factors, including diet and environmental factors, such as vitamin D exposure (Altieri et al., 2017). Chronic pancreatitis resulting from alcohol consumption is also a probable risk factor. Long-term diabetes can increase the risk of pancreatic cancer while also being an early sign of the disease (Klein, 2021). Age and smoking have been recognized as consistent risk factors (Sellam et al., 2015). Traditional Chinese Medicine (TCM) has regarded pancreatic cancer as “fu liang,” “wan tong,” “jaundice,” and “accumulation,” amongst other classifications. Pancreatic cancer is known as the “king of cancers” due to its high degree of malignancy, high rate of recurrence, metastasis, and poor prognosis. The mortality of pancreatic cancer is thus almost equal to its incidence (Zhang et al., 2020).

The specific mechanisms underlying the pathogenesis of pancreatic cancer are poorly understood. Several mutations are reported to be involved, including mutations in Kirsten rat sarcoma (KRAS), Tumor Protein 53 (TP53), cyclin-dependent kinase inhibitor 2A (CDKNA2A), transforming growth factor (TGF), the signaling molecule SMAD4, and cell migration-inducing hyaluronan-binding protein (CEMIP) (Yao et al., 2020; Periyasamy et al., 2022). According to TCM, Qi stagnation and depression of spleen dampness are the main pathogenic factors, with an excess of “dampness, heat, and toxicity” playing a critical role in the onset of pancreatic cancer. Pancreatic cancer has also been associated with liver and gallbladder dysfunction, leading to fluctuations in the functional activity of Qi, and an imbalance between Yin and Yang. This is followed by specific pathological changes in the zang-fu organs, leading to the abnormal secretion and excretion of pancreatic juice, resulting in stagnation and poor eating habits, and ultimately leading to cancer (Deng et al., 2014; Li and Fan, 2016; Wang, L.J., et al., 2016). Western medicine believes that mutations in multiple proto-oncogenes and either the inactivation or deletion of tumor suppressor genes are responsible for cancer development, causing faulty signal transduction and uncontrolled division and proliferation of cells, finally resulting in the formation of a tumor. At the same time, disorders of the internal environment and immune dysfunction contribute to cancer development.

In this review, a comprehensive literature search was conducted using the keywords “Traditional Chinese Medicine (TCM), pancreatic cancer, mechanism, therapy” using Google Scholar (http://scholar.google.com/) and searching the PubMed database (http://www.ncbi.nlm.nih.gov/pubmed). The Chinese websites http://acad.cnki.net/Kns55/brief/result.aspx?dbPrefx=CJFQ from China National Knowledge Infrastructure (CNKI), http://g.wanfangdata.com.cn/ from WanFang, and http://qikan.cqvip.com/from WEIPU, were also searched.

## 2 Current treatment of pancreatic cancer

Currently, Western medicine treats pancreatic cancer mainly by surgical resection, radiotherapy, and chemotherapy. At present, surgical resection is the only possible cure but, as most patients are diagnosed in the middle and late stages, the best time for radical resection is usually missed. Even after surgery, the prognosis may be poor and postoperative recurrence and metastasis are common (Maisonneuve, 2019). Chemotherapy is a systemic treatment and is the most frequently used therapy for middle and advanced pancreatic cancer. However, although cancer cells scattered within tissues or organs can be killed to some extent, the effects of chemotherapy on pancreatic cancer are relatively poor and many researchers believe that pancreatic cancer is resistant to many chemotherapy drugs. In addition, chemotherapy drugs tend to have serious side effects. These factors can render chemotherapy relatively ineffective in treating this cancer. Pancreatic cancer is insensitive to radiotherapy, and, as it is a localized therapy, radiotherapy is not able to effectively control the spread and metastasis of the tumor and improve the patients’ quality of life, so it is rarely used in patients with metastatic pancreatic cancer (Miller et al., 2020). The efficacy of immunotherapy and endocrine therapy is also uncertain (Zhang B et al., 2023). Drugs targeting molecular pathways are one of the research hotspots of tumor therapy, but the combination of the tyrosine kinase inhibitor Erlotinib and the chemotherapy drug gemcitabine, which has been recommended for treating pancreatic cancer, has not been found to significantly improve the quality of life of patients and increases the incidence of side effects such as rash and diarrhea (Schizas et al., 2020). Currently, more attention is being paid to methods of reducing the toxic side-effects of drugs, improving the patients’ quality of life, and prolonging their survival. Many researchers have begun to study the effects of TCM on pancreatic cancer and have made great progress (He et al., 2020; Wang et al., 2022). TCM has many advantages and is being recognized as an indispensable and important facet of comprehensive clinical treatment (Li, B., et al., 2015).

TCM is a medicinal system that has been used for over 2000 years in China, as well as having broad use in other Asian countries. There is currently widespread research into TCM and it has inspired many new discoveries in drug development. One of the basic theories in TCM is that of Yin-Yang balance, which is also applied as a philosophical term. Therein, Yin represents repressive and inhibitory factors while Yang stands for active and aggressive factors. The confrontation, homeostatic balance, and transformation between Yin and Yang comprise the Yin-Yang balance (Yan et al., 2020). Another basic tenet of TCM is the theory of Qi-blood balance. Qi describes the life-force energy and is carried throughout the body via meridians, acting as the conduits of the body energy. Blood represents the source of body energy (Wang et al., 2018). In TCM theory, disturbances in the Yin-Yang balance or Qi deficiency and blood stasis are the underlying causes of disease.

The unique concepts and theoretical foundations of TCM, combined with Western medicine, consider both the whole and the local, host and cancer, and symptoms and disease, with each drawing on the other’s strengths to make up for shortcomings. TCM can not only control tumor development and prolong patient survival but can also improve the immunosuppressant status of the tumor host and reduce the toxic side effects of radiation and chemotherapy, as well as having synergistic effects on other therapies, relieving patients’ pain, and improving their quality of life. Therefore, the study of the treatment of pancreatic cancer with TCM has the potential to provide broader ideas and methods for clinical practice (Li, B., et al., 2015; Yan et al., 2020; Wang et al., 2018; Zhang et al., 2008; Zhang et al., 2010). Some researchers have suggested that Traditional Chinese and Western medicine should be integrated in treating patients with pancreatic cancer that cannot be removed surgically (Yang et al., 2015; Jiang et al., 2019). At the beginning of treatment, radiotherapy, and chemotherapy, supplemented by TCM, should be the main treatment methods, acting as the “first attack” on the tumor cells, followed by long-term maintenance treatment with TCM, which can delay the progression of the tumor and improve the long-term survival rate of the patients (Schizas et al., 2020).

## 3 Treatment with Chinese herbs

There has been extensive investigation into the role of Chinese medicine in the treatment of pancreatic cancer, with many studies showing that TCM can improve the survival of patients with pancreatic cancer (Kuo et al., 2018; Wong et al., 2019). TCM has many advantages, including fewer toxic side-effects, pain relief, and improvement in the quality of life of patients. In this article, we summarize some TCM and their mechanisms of action in the treatment of pancreatic cancer.

### 3.1 Compound traditional Chinese medicine

TCM focuses on macroscopic and external phenomena, such as external clinical manifestations and the adjustment of the integrity of the human internal environment. Western medicine, in contrast, focuses on microscopic and internal mechanisms, such as precise targeting and reducing the size of the tumor (Wang et al., 2018). The composition of TCM is complex, and researchers are attempting to understand its underlying mechanisms from the perspective of Western medicine. Table 1 summarizes the effects and possible mechanisms of several TCM compounds in regulating pancreatic cancer.

**TABLE 1:** The main mechanisms of action of TCM compounds on pancreatic cancer.

Types of TCM	Test system	Biological effects	Refs
Qingyi Huaji (QYHJ) herbal decoction	Patients, mouse, SW1990 cells	Inhibits tumor cell growth and metastasis	Ouyang et al. (2010), Wang P et al. (2010), Xu Y L et al. (2015), Song et al. (2017), Song et al. (2018), Chen et al. (2019), Qian et al. (2020)
Wei Tiao San Hao decoction	Patients	Promotes tumor apoptosis, enhances immune function, inhibits tumor cell growth and metastasis	Ni et al. (2006), Ni et al. (2013a), Ni et al. (2013b), Xue et al. (2016)
Phenolic alkaloids of *Menispermum dauricum*	Patients, mouse, BxPC-3 cells	Promotes tumor apoptosis, enhances immune function, inhibits tumor cell growth and metastasis	Su et al. (2007), Meng et al. (2010), Wei et al. (2015), Zhou Z G et al. (2015), Wu et al. (2019)
Ezhukuijian Decoction	Mouse, MiaPaCa-2 cells	Promotes tumor apoptosis, enhances immune function, inhibits tumor cell growth and metastasis	Bu et al. (1999)
Yin Chen Hao Decoction	Patients, PANC-1 cells	Promotes tumor apoptosis	Zhou H B et al. (2015)
Gexia Zhuyu Decoction or Liujun Ermu Decoction	Patients	Increases overall survival	Li M et al. (2018)
Wumei Pill	Patients	Alleviate symptoms	Wan et al. (2019)
Aidi Injection	Mouse	Inhibits tumor cell growth	Wang H et al. (2023)

#### 3.1.1 Qingyi Huaji (QYHJ) herbal decoction

The QYHJ formula contains many traditional Chinese medicines, including *Rhizoma Amorphophalli*, *Oldenlandia*, *Scutellaria barbata, Gynostemma pentaphyllum*, and *Amomum cardamomum*. The QYHJ herbal decoction reduces heat and resolves toxicity, regulating the Qi and dispelling dampness, and can stabilize the focus and inhibit the spread of the tumor. Song LB et al. reviewed 232 cases of postoperative pancreatic cancer patients treated with QYHJ combined with Western medicine and found that taking QYHJ for more than 3 months could prolong postoperative survival and improve long-term survival (Song et al., 2018). It was found the levels of cytokines, including vascular endothelial growth factor (VEGF), tumor necrosis factor-*α* (TNF-*α*), soluble vascular cell adhesion molecule (sVCAM-1), transforming growth factor-*β*1 (TGF-*β*1), interleukin 6 (IL-6), and interleukin 8 (IL-8), were significantly increased in the sera of pancreatic cancer patients. One of the mechanisms responsible for the effects of QYHJ in the treatment of pancreatic cancer may be to downregulate the levels of TNF-*α*, sVCAM-1, TGF-*β*1, IL–6, and IL-8 in the serum, together with inhibiting several signal transduction pathways, thus improving the immunity of patients and inhibiting the growth and metastasis of tumor cells (Ouyang et al., 2010). Previous studies have shown that QYHJ can downregulate the expression of the *Ski* gene, restore the functions of Notch-4 and Jagged-1, and prolong the survival of patients with advanced pancreatic cancer (Wang P et al., 2010; Xu Y L et al., 2015; Song et al., 2017). QYHJ can also reverse the drug resistance of pancreatic cancer to gemcitabine, possibly through inhibiting the differentiation of pancreatic cancer stem cells through the lncRNA AB209630/miR-373/EphB2 NANOG signaling pathway (Chen et al., 2019). Using network pharmacology and weighted gene co-expression network analysis (WGCNA), we found that CDK1, PLD1, MET, F2RL1, XDH, NEK2, TOP2A, NQO1, CCND1, PTK6, CTSE, and ERBB2 may be reliable immune-related biomarkers for the prediction of pancreatic cancer prognosis, and may be important immunotherapeutic targets of QYHJ (Qian et al., 2020).

#### 3.1.2 Wei Tiao San Hao decoction (WD-3)

WD-3 mainly includes *Codonopsis pilosula*, *Rhizoma atractylodes*, *Macrocephalae, Poria cocos* with hostwood, Rhizoma Pinelliae pericarpium Citri reticulatae, Agaric, caulis perllae, Fructus Aurantii immaturus, coix seed, Rhizoma dioscoreae, rice-grain sprout, Fructus hordei germinates, Mongolian snakegourd, Radix cynanchi panicullati, and *Akebia trifoliata* Koidz. This method is based on “Zhao’s fine-tuning balance theory” established by the oncologist Zhao Jingfang who believes that the development of cancer is due to dysregulation of the body’s internal environment, and dysfunction of the viscera, Yin and Yang, Qi, and blood disorders. To treat tumors, the key point of the imbalance should be determined, specifically, whether the spleen and stomach in the middle-jiao are dysfunctional, and make adjustments to restore the balance of the body to restore immune function, allowing control of the disease and improvement of the quality of life and long-term survival of patients. Therefore, the treatment of middle-jiao focuses on the regulation of the Qi and the blood. This strengthens the spleen and moistens and harmonizes the spleen and stomach, while assisting healthy Qi, regulating Qi, and eliminating accumulation, balancing Yin and Yang, Qi, and the blood, and, at the same time, reducing abdominal discomfort, jaundice, poor appetite, fatigue, and other clinical symptoms. This can not only inhibit tumor development but can also increase the efficacy of chemotherapy, reduce toxic side effects, improve the quality of life of patients, and prolong their lives. From the perspective of modern pharmacological mechanisms, WD-3 mainly regulates the functioning of the immune system and homeostasis in the body, inhibiting the growth and metastasis of the tumor by increasing the levels of cytokines IL-2, IL-4, and IFN-*γ* in the body and enhancing the function of immune cells. In addition, some components such as semen coicis can halt the cancer cell cycle in the G2/M phase, inhibiting the proliferation and promoting the apoptosis of cancer cells (Ni et al., 2006; Ni et al., 2013a; Ni et al., 2013b; Xue et al., 2016).

#### 3.1.3 Phenolic alkaloids of *Menispermum dauricum (*PAMD)

PAMD is a mixture extracted from *Menispermum dauricum*, containing a variety of fat-soluble alkaloids and is mainly composed of dauricine (Dau) and daurisoline (DS) (Su et al., 2007). PAMD can inhibit tumor angiogenesis by downregulating the expression of VEGF and bFGF in tumor tissues of xenografts of the BxPC-3 human pancreatic cell line in nude mice, causing tumor cell necrosis, inhibiting tumor growth, and inducing a corresponding inflammatory response. Previous studies have shown that PAMD can significantly inhibit the growth of tumors in BxPC-3 mice and enhance apoptosis of BxPC-3 cells by promoting the secretion of TNF-*α* (Meng et al., 2010; Zhou Z.G et al., 2015). Relevant studies have shown that imbalances in or abnormal activation of the Hedgehog signaling pathway are closely related to the occurrence of pancreatic cancer and its biological characteristics. PAMD may influence the expression of Gli1mRNA, a key molecule in the Hedgehog signaling pathway, inhibiting the proliferation of the BxPC-3 cells (Wei et al., 2015; Wu et al., 2019). PAMD can also regulate the body’s immune function, improve the biological activity of cytokines, downregulate the expression of K-Ras mRNA, upregulate the expression of DPC4 mRNA, and activate the TGF-*β* signaling pathway, leading to the inhibition of tumor growth and proliferation (Feng et al., 2010; Wang J M et al., 2010; Zhong et al., 2014; Liu et al., 2015; Qiu et al., 2016).

#### 3.1.4 Ezhukuijian decoction

The main components of the Ezhukuijian decoction are *Curcuma zedoary, Rhizoma corydalis*, peach kernel, and bupleurum. Studies have shown that bupleurum is principally responsible for the anti-tumor effect (Cai et al., 2023). Saikosaponin (SS) is the main active component of bupleurum, which can significantly inhibit the proliferation of pancreatic cancer cells. There are nine kinds of SS of which saikosaponin-d (SSd) has the strongest pharmacological activity. SSd may inhibit the MAPK signaling pathway and reduce the expression of Dcr3, thus inhibiting the proliferation and metastasis of pancreatic tumor cells and promoting their apoptosis. SSd has a glucocorticoid-like steroid ring structure and has also been found to regulate the immune function of the body through various mechanisms, significantly increasing the phagocytic activity of macrophages, increasing the activity of T-lymphocytes, and inducing the expression of IL-2 and its receptor, thereby enhancing the specific and non-specific immune response of the body (Bu et al., 1999).

#### 3.1.5 Yin Chen Hao Decoction (YCHD)

Yin Chen Hao Decoction (YCHD), a classic Chinese medicinal formula consisting of three herbal drugs, *Rheum officinale* Baill, *Artemisia capillaris* Thunb, and *Gardenia iasminoides* Ellis, is a potent inhibitor of carcinoma. YCHD induces the apoptosis of PANC-1 cells, mediated in part via upregulation of BAX and downregulation of Bcl-2 (Zhou H B et al., 2015).

#### 3.1.6 Gexia Zhuyu Decoction or Liujun Ermu Decoction

Gexia Zhuyu Decoction is a prominent formula for treating blood stasis syndrome. Liujun Ermu Decoction is an effective prescription for treating phlegm dampness syndrome. Both decoctions have been found to increase the overall survival of pancreatic tumor patients. In a study (Li M et al., 2018), 174 patients were allocated to groups that received comprehensive medicine (IM) or TCM, according to whether they received treatment with Western medicine. The patients were treated with Gexia Zhuyu Decoction or Liujun Ermu Decoction, according to syndrome differentiation, twice a day for at least 2 months, after which their overall survival (OS) was compared. In the I/II phase, the median OS was found to be 20.5 months in the IM group (95% confidence interval [CI], 12.499–28.501) and 11.17 months in the TCM group (95% CI, 5.160 to 17.180, *p* = 0.015). The 1-year and 2-year survival rates were 47.0% and 40.0%, respectively, for the IM group and 21.0% and 21.0%, respectively, for the TCM group. In Phase III/IV, the median OS was 13.53 months (95% CI, 8.665–18.395) in the IM group and 6.4 months (95% CI, 0.00–15.682) in the TCM group (*p* = 0.32). The 1-year and 2-year survival rates in the IM group were 27.0% and 7.0%, respectively, while those in the TCM group were 20.0% and 2.0%, respectively. Thus, TCM intervention contributed to the survival of pancreatic patients at different stages (Li M et al., 2018).

#### 3.1.7 Other herbal formulations

Wumei Pill (WMP) is a well-known herbal formula in China and has been used in clinical practice for the treatment of digestive system disorders for hundreds of years. Wan et al. found that WMP can alleviate symptoms of pancreatic cancer through molecular mechanisms predicted by network pharmacology (Wan et al., 2019). Aidi Injection (ADI) is an anti-tumor herbal medicine that is administered by injection, and can inhibit the growth of pancreatic cancer by acting on VEGFA, P53, CASP3, and JUN in tumor-bearing animals (Wang G et al., 2023).

### 3.2 Active components of Chinese traditional herbs

**FIGURE 1:**
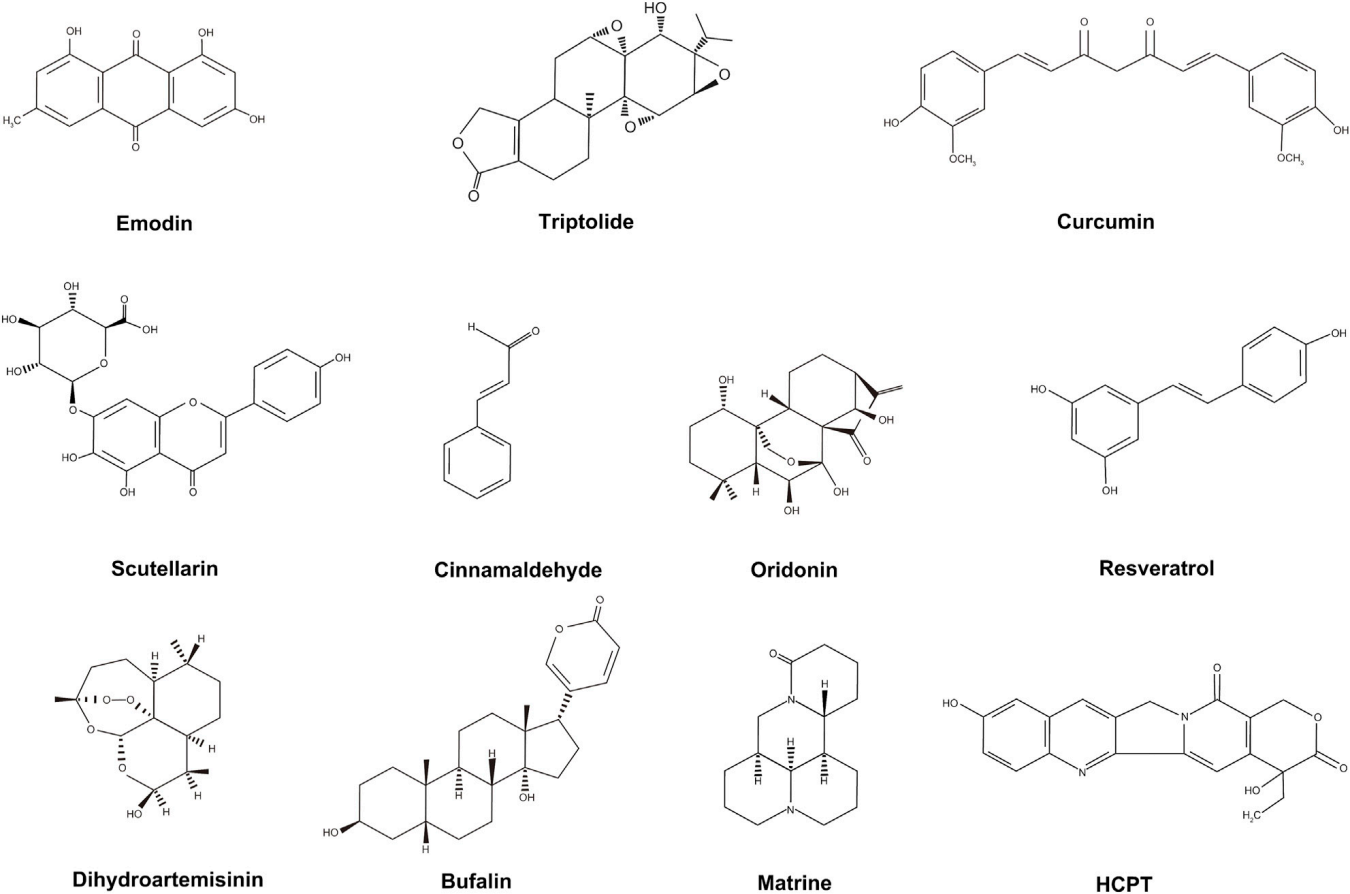
Structures of identified TCM active components.

Many of the active components of TCM are also regarded as effective for the treatment of pancreatic cancer, including emodin, triptolide, and curcumin. The structures of these active components are shown in Figure 1. These active TCM components appear to benefit the treatment of pancreatic cancer mainly by inhibiting proliferation and promoting apoptosis of cancer cells; however, to date, their effects have only been investigated in animal models or pancreatic cancer cells *in vitro*. The details are shown in Table 2.

**TABLE 2:** Mechanisms of TCM active components on pancreatic cancer.

Types of TCM monomer	Pubchem ID	Test system	Signaling	Refs
EMO	3220	Mouse, pancreatic cancer lines (PANC-1 BxPC-3, MIAPaCa-2, SW 1990, ASPC-1)	JAK/STAT Signaling	Guo et al. (2009), Guo et al. (2012), Zhang et al. (2013), Yao et al. (2015), Li, N.,et al. (2018), Liu, D.L.,et al. (2020), Tong et al. (2020)
			PI3K signaling	
			TGF -β signaling	
			Notch Signaling	
			Fas/Fasl signaling	
TPL	107,985	Mouse, pancreatic cancer lines (PANC-1, BxPC-3, MIAPaCa-2, SW 1990, ASPC-1, Capan-1, Capan-2, SNU-213, SNU-410, HPAFII, and Hs766T)	NF-*κ*B signaling	Phillips et al. (2007), MacKenzie et al. (2013), Ding et al. (2015), Zhang et al. (2017), Subramaniam et al. (2018), Dai et al. (2019), Feng et al. (2019), Ma J X et al. (2019)
			Sonic hedgehog signaling	
			Fas/Fasl signaling	
Curcumin	969,516	Mouse, pancreatic cancer lines (PANC-1 BxPC-3, MIAPaCa-2, SW 1990, ASPC-1, SNU-410, and Hs766T)	JAK/STAT Signaling	Glienke et al. (2010), Nagaraju et al. (2015), Cao et al. (2016b), Ning et al. (2016), Wang Q., et al. (2017), Zhu and Bu (2017), Li W., et al. (2018), Li W., et al. (2019), Nagaraju et al. (2019), Li W., et al. (2020)
			PI3K signaling	
			TGF -β signaling	
			NF-*κ*B signaling	
			Sonic hedgehog signaling	
			Fas/Fasl signaling	
Scutellarin	185,617	Mouse, pancreatic cancer line (PANC-1)	Hippo-YAP signaling	Cai et al. (2017), Wang, L., et al. (2019), Chen et al. (2022)
Cinnamaldehyde	637,511	Pancreatic cancer line (BxPC-3)	Fas/Fasl signaling	Lv et al. (2012)
Ori	5321010	Mouse, pancreatic cancer lines (PANC-1, BxPC-3, SW 1990, MIAPaCa-2, ASPC-1)	NF-*κ*B signaling	Bu et al. (2014), Liu D.L., et al. (2014), Xu B et al. (2015), Chen et al. (2014), Liu Q Q et al. (2016), Gui et al. (2015), Liu, D.L., et al. (2020), Zhao et al. (2021)
			Fas/Fasl signaling	
Res	445,154	Mouse, pancreatic cancer lines (PANC-1 BxPC-3, MIAPaCa-2, ASPC-1, Capan-2, Colo357, and Hs766T)	Hippo-YAP signaling	Li, W., et al. (2013), Hoca et al. (2020), Ma J G et al. (2019), Chowdhury et al. (2018), Duan et al. (2016), Zhu et al. (2016), Li, W., et al. (2016), Cao et al. (2016a), Jiang et al. (2016), Subramaniamet al. (2018), Florio et al. (2023)
			PI3K signaling	
			NF-*κ*B signaling	
			Sonic hedgehog signaling	
DHA	3000518	Mouse, pancreatic cancer lines (PANC-1, BxPC-3, MIAPaCa-2, SW 1990, ASPC-1, CFPAC-1)	NF-*κ*B signaling	Chen, H., et al. (2009), Kong et al. (2012), Wang, S.J., et al. (2011), Wang S J et al. (2010), Li, Y.L., et al. (2016), Hu et al. (2020), Du et al. (2021), Zhang Q et al. (2022)
			Fas/Fasl signaling	
Bufalin	9547215	Mouse, pancreatic cancer lines (PANC-1, BxPC-3, MIAPaCa-2, SW 1990, CFPAC-1, CFPAC-2)	JAK/STAT Signaling	Chen, Y., et al. (2012), Li, M.Y., et al. (2014), Liu X et al. (2016), Ning, Z.Y., et al. (2019), Wang, H.Y., et al. (2016), Liu X et al. (2020), Fu et al. (2021), Yu et al. (2022)
			NF-*κ*B signaling	
			Sonic hedgehog signaling	
			Fas/Fasl signaling	
Matrine	91,466	Mouse, pancreatic cancer lines (PANC-1, MIAPaCa-2, PaTu 8988t, BxPC-3, Capan-1, HPAC)	Wnt/β catenin signaling	Liu, T.Y., et al. (2010), Cho et al. (2018), Huang and Xin (2018), Ma, Y.C., et al. (2015), Zhuang et al. (2020)
			Fas/Fasl signaling	
HCPT	97,226	Mouse, pancreatic cancer lines (PANC-1, Capan-1, BxPC-3**)**	NF-*κ*B signaling	Yang et al. (2011), Zhang et al. (2019), Zhong et al. (2023)
			Fas/Fasl signaling	

#### 3.2.1 Emodin

Emodin (EMO, 6-methyl-1, 3, 8-trihydroxy anthraquinone), derived from rhubarb, *Polygonum*, *Prunus*, and folium sennae, has many biological activities and acts as an inhibitor of tyrosine kinase Ⅱ (Zhang H B et al., 2022). Emodin significantly inhibits the proliferation of pancreatic cancer SW1990 cells and induces apoptosis. Studies have found that emodin induced apoptosis of pancreatic cancer through inhibiting the activity of NF-*κ*B, downregulating the expression of Bcl-2 and survivin, and upregulating the expression of BAX, thus reducing the Bcl-2/BAX ratio (Tong et al., 2020). In addition, emodin treatment resulted in the downregulation of NF-*κ*B, together with its regulatory factors VEGF and MMPs, as well as Survivin, inhibiting angiogenesis in pancreatic cancer, and thus inhibiting the growth and metastasis of the cancer. Emodin can also enhance the anti-tumor effect of gemcitabine on SW1990 transplantation tumors, possibly by preventing the activation of Akt and NF-*κ*B in pancreatic cancer tissue, reducing the expression of Bcl-2, and upregulating the expression of BAX. The resultant decrease in the Bcl-2/BAX ratio reduced mitochondrial damage and increased the permeability of the mitochondrial membrane. CytC was then released from the mitochondria, activating caspase-3 and caspase-9 and promoting apoptosis (Guo et al., 2009; Guo et al., 2012; Zhang et al., 2013; Yao et al., 2015). Li found that emodin can also inhibit the metastasis and invasion of pancreatic cancer SW1990 cells through various mechanisms (Li N et al., 2018) while Liu et al. demonstrated that emodin may increase the expression of Bid in SW1990 cells and may change the physiological environment of the mitochondria by reducing the mitochondrial membrane potential, thus promoting cell apoptosis and inhibiting cell proliferation (Liu D L, et al., 2020). Pan et al. found that emodin combined with 5-Aza-CdR enhanced the demethylation of the tumor suppressor gene p16, RASSF1A, and ppENK by reducing the expression of the methyltransferases DNMT1 and DNMT3a (Pan et al., 2016). Wei et al. demonstrated that EMO reverses gemcitabine resistance in pancreatic cancer by inhibiting stem cells (Wei et al., 2022). EMO was also found to enhance immunity by regulating the ratio of T helper type 1 (TH1), TH2, TH17, and γδ T cells, as well as interferon γ/interleukin 17-producing γδ T cells (Zhou et al., 2021).

#### 3.2.2 Triptolide (TPL)

Triptolide, a diterpene triepoxide, is the main active ingredient of Tripterygium, which has the functions of expelling wind-damp, smoothing collaterals, relieving pain, and detoxification (Wang G et al., 2023). Its chemical properties are stable *in vivo*, and its titer is 100–200 times higher than that of *Tripterygium wilfordii* Hook f. (TWHF). Triptolide has many biological activities, and its anti-tumor effect has been widely investigated in recent years (Mu et al., 2022). Triptolide shows broad-spectrum anti-tumor activity. Triptolide is known to inhibit the growth and proliferation of more than 60 types of tumor cells. This inhibitory effect may be achieved by blocking the cell cycle, inducing tumor cell apoptosis, and inhibiting tumor cell proliferation and metastasis. Triptolide is also effective for tumor cells that have already developed drug resistance and has a synergistic effect when combined with chemotherapy drugs, such as gemcitabine, and radiotherapy. It can not only reduce the occurrence of drug resistance but can also significantly enhance tumor cell apoptosis induced by radiotherapy and chemotherapy and inhibit tumor cell proliferation (Zhang et al., 2017). Some of the anti-pancreatic cancer mechanisms of triptolide may be: ①Increasing the production of reactive oxygen species (ROS), thus damaging the cell’s DNA and mitochondria and upregulating the expression of the pro-apoptotic protein BAX and NF-*κ*B, which, in turn, downregulate the expression of Bcl-2, and increasing the permeability of the mitochondrial membrane to induce apoptosis (Ma J X et al., 2019). ②HSP70 is a heat shock protein and chaperone, which can protect cells from oxidative stress and other damage. It is expressed at low levels in normal cells while showing strong expression in various tumor cells, and has been shown to inhibit apoptosis through various mechanisms and participate in the development of tumors. Triptolide can significantly downregulate the expression of HSP70, leading to cell death (Phillips et al., 2007; MacKenzie et al., 2013). ③ Inhibiting the ball-forming and tumorigenic ability of pancreatic cancer stem cells (Liu L et al., 2014; Subramaniam et al., 2018).④ Inhibiting activation of the Hedgehog signaling pathway, thereby inhibiting the growth of various pancreatic cancer cells and inducing apoptosis (Feng et al., 2019). ⑤ Activating autophagy via downregulation of Pumilio RNA-binding family member 1 (PUM1) (Dai et al., 2019).⑥ Suppressing proliferation, through downregulation of the hypoxia-inducible factor-1*α* and c-myc expression in pancreatic cancer cells (Ding et al., 2015).

#### 3.2.3 Curcumin

Turmeric is obtained from plants of the genus *Curcuma* and its main components are essential oil, fat oil, and curcumin, with the most active component being curcumin (C_21_H_20_O_6_), a *β*-diketone polyphenolic compound. Curcumin is used as a non-steroidal anti-inflammatory drug and has the effect of breaking blood, promoting Qi, and relieving pain through the meridional circulation (Huang et al., 2022). The anti-pancreatic cancer mechanism of curcumin may include: ① Inhibiting cell migration (Li, W., et al., 2019; Li W et al., 2018; Nagaraju et al., 2019); ② Downregulating the expression of Bcl-2 and upregulating the expression of BAX, decreasing the Bcl-2/BAX ratio, and thus inducing apoptosis (Zhu and Bu, 2017); ③Inhibiting the epithelial-mesenchymal transformation of pancreatic cancer PANC-1 cells induced by TGF-*β*1, thereby reducing the possibility of invasion and distant metastasis (Wang, Q., et al., 2017; Cao et al., 2016b; Li W, et al., 2020); ④ Counteracting angiogenesis through reducing the activity of COX-2, preventing the synthesis of PGE2 and thus inhibiting tumor formation (Nagaraju et al., 2015); ⑤ Inhibiting pancreatic cancer stem cells (Ning et al., 2016); ⑥ Inhibiting the phosphorylation of STAT3 in pancreatic cancer cell lines, thus blocking STAT3 activation, and promoting apoptosis by enhancing the cytotoxicity of natural killer (NK) cells (Glienke et al., 2010; Fiala, 2015; Malhotra et al., 2022); ⑦ Inhibiting the growth of xenograft tumors and the biological activity of pancreatic cancer cells by regulating the miR-21–5p/SMAD7 axis (Liu et al., 2012; Fang et al., 2022).

#### 3.2.4 Scutellarin

*Scutellaria barbata* (family Lamiaceae), with its principal active component scutellarin, has the effect of clearing heat and detoxification, as well as dissolution of stagnancy and diuresis. The plant has many pharmacological activities, including anti-tumor, antioxidant, and anti-bacterial effects, and can induce cell apoptosis and inhibit tumor angiogenesis. In combination with other Chinese herbs, it is effective for treating various cancers including pancreatic cancer (Cai et al., 2017; Wang, L., et al., 2019). Cai Yunyun et al. found that the expression of YAP protein, a key factor of the Hippo signaling pathway, was reduced in nude mice treated with *S. barbata* extract, while the expression of phosphorylated YAP was increased, inhibiting the proliferation, invasion, and metastasis of pancreatic cancer cells (Cai et al., 2017). Scutellarin and its analogs can be used as adjuvants to enhance the anti-tumor effect of immunotherapeutic agents by inhibiting TNFR2+Treg activity (Chen et al., 2022).

#### 3.2.5 Cinnamaldehyde

Cinnamaldehyde is a monomeric component extracted from the Chinese traditional medicine cassia twig and cinnamon, and has the effects of warming and assisting Yang. Cinnamaldehyde has many effects, including antipyretic, antiviral, and anti-cardiovascular, as well as anti-tumor properties and promoting fibroblast proliferation. Studies have shown that cinnamaldehyde can promote the apoptosis of BxPC-3 pancreatic cancer cells *in vitro*, inhibiting their growth and inhibiting metastasis to some extent. It may induce cancer cell apoptosis by upregulating the expression of Bcl-2 and pro-caspase-9 proteins and activating the mitochondrial apoptosis signaling pathway (Lv et al., 2012).

#### 3.2.6 Oridonin

Oridonin (Ori), a kauri diterpene isolated from *Rabdosia rubescens*, has many biological effects, including anti-inflammatory, antibacterial, and anti-tumor properties (Qiu et al., 2018). The anti-tumor activity is mainly due to inhibiting the proliferation of tumor cells and inducing tumor cell apoptosis. The molecular mechanisms underlying the action of Ori against pancreatic cancer are as follows: ①Downregulating the expression of survivin in cells, thus reducing its inhibitory effects on caspase-3 and caspase-7, and inducing apoptosis of cancer cells, together with upregulating the expression of p21, arresting the cell cycle in the G2/M phase, and inhibiting the proliferation of cancer cells (Bu et al., 2014); ② Downregulating the expression of Bcl-2 and upregulating the expression of BAX, followed by activation of the caspase signaling pathway to induce apoptosis (Liu D L et al., 2014; Liu D L et al., 2020); ③Inducing DNA damage in pancreatic cancer SW1900 cells and phosphorylation of H2AX protein, resulting in increased intracellular *γ*-H2AX content (Xu B et al., 2015); ④ Inhibiting the NF-*κ*B and MAPK signaling pathways and silencing STAT3, thus blocking the STAT3 signal transduction pathway, thereby inhibiting pancreatic cancer cell growth (Chen et al., 2014); ⑤Regulating the epithelial-mesenchymal transition (Liu Q Q et al., 2016; Lou et al., 2019); ⑥Inducing autophagy-mediated cell death in pancreatic cancer (Zhao et al., 2021); ⑦ Regulating microRNAs or decreasing Treg differentiation (Gui et al., 2015; Guo et al., 2020).

#### 3.2.7 Resveratrol (Res)

Res, 3,4, 5-trihydrox-1, 2-distyrene, a non-flavonoid polyphenolic compound with the molecular formula C_14_H_12_O_3_, is widely found in various plants and has antioxidant and anti-tumor effects, as well as promoting cardiovascular protection (Grau et al., 2023; Xie et al., 2023). Its anti-tumor activity may be achieved by inhibiting the tumor cell cycle, promoting apoptosis, and inhibiting invasion, metastasis, and angiogenesis. Specifically, these effects may be the result of ① Regulation of the epithelial-mesenchymal transition through suppression of PI3K/AKT/NF-*κ*B (Li, W., et al., 2013; Hoca et al., 2020), ②Inhibition of proliferation through inhibition of MAPK/ERK and HIF-1*a* (Chowdhury et al., 2018; Ma J G et al., 2019), ③ Promotion of apoptosis through inhibition of VEGF, STAT3, and Bcl-2 (Duan et al., 2016; Zhu et al., 2016), ④ Inhibition of invasion and migration through inhibition of Up, E-cadherin, and Hedeghog (Li, W., et al., 2016; Cao et al., 2016a), ⑤ Increasing sensitivity to chemotherapy drugs through upregulation of YAP (Jiang et al., 2016), and ⑥ Inhibition of pancreatic cancer stem cells (Subramaniamet al., 2018; Florio et al., 2023). Resveratrol has been found to induce the expression of anti-cancer cytokines such as IFN-γ and TNF- α. It can also stimulate the polarization of CD4^+^ T cells and macrophages towards anti-cancer cells and reduce the infiltration and polarization of immunosuppressive cells (Chen and Musa, 2021).

#### 3.2.8 Dihydroartemisinin (DHA)

DHA is a derivative of artemisinin and is the main active metabolite of artemisinins *in vivo*. In addition to its antimalarial effects, DHA also has anti-tumor effects (Wang Y et al., 2023), which may occur through ① Promotion of apoptosis through downregulating the expression of proliferating cell nuclear antigen and cyclin D1, regulating p21 (WAF1/CIP1), reducing the Bcl-2/BAX ratio, decreasing Mcl-1 expression, and increasing the activation of caspase-9 or DR5 (Chen, H., et al., 2009; Kong et al., 2012; Hu et al., 2020), ② The promotion of angiogenesis through downregulating the expression of the NF-*κ*B-targeted proangiogenic gene products VEGF, IL-8, COX-2, and MMP-9 (Wang, S.J., et al., 2011), ③Inhibition of proliferation through downregulation of NF-*κ*B (Wang S J et al., 2010), and ④Suppression of pancreatic cancer cell growth via a microRNA-mRNA regulatory network (Li, Y.L., et al., 2016). ④Dihydroartemisinin inhibits the growth of pancreatic cells by inducing ferroptosis and activating antitumor immunity (Du et al., 2021; Zhang Q et al., 2022). Zhang et al. found that DHA significantly decreased the suppressive expansion of M2-type macrophages (M2) and myeloid-derived suppressor cells (MDSCs). Moreover, DHA increased the proportions of CD8+T, NK, and NKT cells in the tumor tissues of tumor-bearing mice (Zhang H B et al., 2022).

#### 3.2.9 Bufalin

Bufalin (C_24_H_34_O_4)_ is a bufonterene and is a toxic compound extracted from toad venom. It has digoxin-like immune-stimulatory activity and extensive anti-tumor activity, which may result from ① The induction of apoptosis through cell cycle arrest, inhibition of the activity of Hsp27, direct activation of pro-caspase-3, caspase-8, and pro-caspase-9, as well as increasing the expression of ASKI1/JAK, NF-*κ*B, and reducing the Bcl-2/BAX ratio (Chen Y., et al., 2012; Li M.Y., et al., 2014; Liu X., et al., 2016; Ning Z.Y., et al., 2019; Liu X et al., 2020) and ②Suppression of cancer stem-like cells in gemcitabine-resistant pancreatic cancer via Hedgehog signaling (Wang and Lu, 2016). In addition, Bufalin was found to enhance the killing efficacy of NK cells and to stimulate the anti-tumor immune response by driving tumor-infiltrating macrophage toward the M1 phenotype (Fu et al., 2021; Yu et al., 2022).

#### 3.2.10 Matrine

Matrine is a tetracyclic quinazine compound with the molecular formula C15H24N20. It is an active alkaloid widely found in leguminous plants (Zhang M W et al., 2023). It has extensive pharmacological effects, including antiviral, anti-inflammatory, anti-fibrosis, and anti-tumor activities (Xu et al., 2022). Matrine can play an anti-pancreatic cancer effect through various mechanisms: ①Inducing apoptosis by reducing the Bcl-2/BAX ratio, upregulating Fas, and increasing activation of caspases-8, -3 and -9, inhibiting mitochondrial energy production (Liu, T.Y., et al., 2010; Cho et al., 2018); ②Inhibiting pancreatic cancer cell migration and invasion through the ROS/NF-κB/MMPs pathway (Huang and Xin, 2018); ③ Inhibiting HPAC cellular migration and invasion through down-regulating the expression of MT1-MMP via the Wnt signaling pathway (Ma, Y.C., et al., 2015). Matine suppresses macrophage-mediated immunosuppression and subsequently upregulates CD8^+^ T cell cytotoxic activities (Zhuang et al., 2020).

#### 3.2.11 Hydroxycamptothecin (HCPT)

HCPT ((S)-4,9-dihydroxy-4-ethyl-1h-pyran [3′, 4′: 6,7] indene [1.2b] quinoline-3,14-(4H, 12H)-Dione is a cell cycle-specific drug that induces apoptosis through cell cycle arrest. It has been found that HCPT has an inhibitory effect on the growth of pancreatic cancer cells, possibly through activation of caspase-9/caspase-3 and inhibition of the NF-*κ*B signaling pathway (Yang et al., 2011; Zhang et al., 2019; Zhong et al., 2023).

## 4 Challenges and future perspectives

**FIGURE 2:**
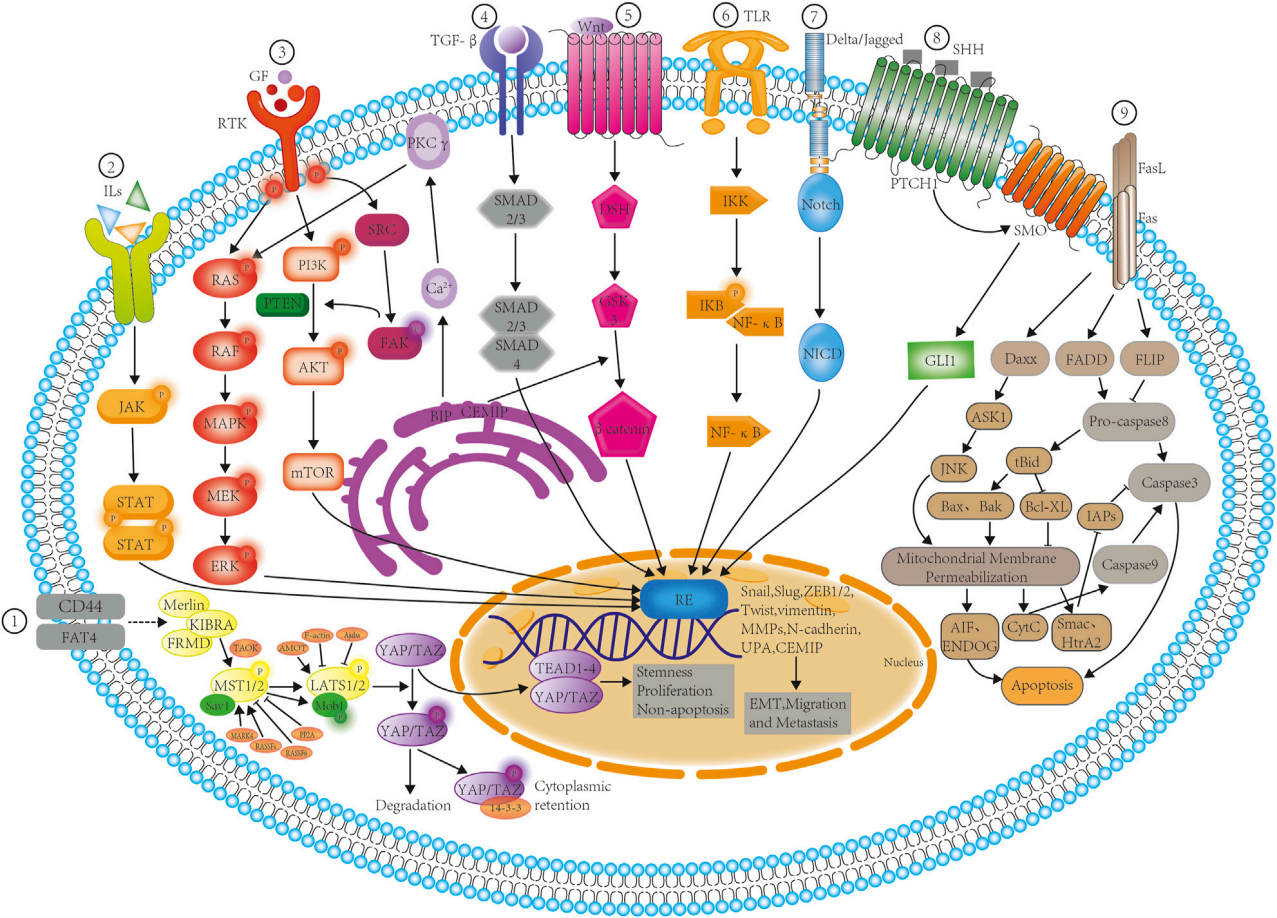
TCM inhibition of signaling pathways involved in the EMT, migration, metastasis, and apoptosis of pancreatic cancer cells. The schematic diagram shows the various TCM formulations involved in the inhibition of the nine key signaling processes as follows: ① Via Hippo-YAP signaling, involving Scutellarin and Res; ② Via JAK/STAT signaling, involving EMO, Curcumin, and Bufalin; ③ Via tyrosine kinase receptor signaling, involving EMO, Curcumin, and Res; ④ Via TGF -β signaling, involving EMO and Curcumin; ⑤ Via Wnt/β catenin signaling, involving Curcumin and matrine; ⑥ Via NF-κB signaling, involving TPL, Curcumin, Ori, Res,DHA, Bufalin, and HCPT; ⑦ Via Notch signaling, involving EMO; ⑧ Via sonic hedgehog signaling, involving TPL, Curcumin, Res, and Bufalin; ⑨ Via Fas/Fasl signaling, involving EMO,TPL, Curcumin, Cinnamaldehyde, Ori, DHA, Bufalin, matrine, and HPCT. Abbreviations: DSH- phosphoprotein Dishevelled; ERK- Extracellular signal-regulated kinases; FAK- Focal adhesion kinase; GF- Growth factor; GLI1- Glioma-associated oncogene homologue 1; GSK3- Glycogen synthase kinase 3; IKK- IκB kinase; IL- Interleukin; IκB -Inhibitor of nuclear factor kappa; JAK- Janus kinase; MAPK- Mitogen-activated protein kinase; MEK- MAPK/Erk kinase; MMP- matrix metalloproteinase; mTOR-mechanistic target of rapamycin; NF-κB- nuclear factor kappa-light-chain-enhancer of activated B cells; NICD- Notch intracellular domain; PI3K- phosphatidylinositol-3 kinase; PKC- Protein kinase C; PTCH1- Protein patched homolog 1; RAF- Rapidly Accelerated Fibrosarcoma; RAS- Rat sarcoma; RE- Response elements; RTK- Receptor tyrosine kinases; SHH- Sonic hedgehog; SMO- Smoothened protein; STAT-signal transducers and activators of transcription; TGF-β- Tumor growth factor-beta; TLR- Toll-like receptor; UPA-urokinase plasminogen activator; ZEB- Zinc finger E-box binding homeobox 1; YAP- Yes associated protein; TAZ- Transcriptional coactivator with PDZ-binding motif.

In recent years, great progress has been made in the investigation of TCMs for the treatment of pancreatic cancer. TCM shows unique advantages in the treatment of pancreatic cancer, not only by controlling tumor progression and prolonging the survival of patients, but also enhancing immunity, reducing the toxic side effects of radiotherapy and chemotherapy, relieving pain, and improving the patient’s quality of life. A review of the actions of TCM (Table 2) shows that these formulations act via different signaling mechanisms (Figure 2), leading to the inhibition of migration and metastasis and the promotion of apoptosis in pancreatic cancer cells. However, TCM formulations function holistically, as each contains a number of ingredients, which presents a challenge for the establishment of quality control standards for the raw materials and the standardization of the final herbal drugs as no single component is directly responsible for the total efficacy (Peng et al., 2018). The concept of TCM chemical fingerprints aiming to obtain a comprehensive characterization of these complex chemical matrices is one of the most convincing tools for the quality assessment of TCM. (Li, Y., et al., 2020; Ma et al., 2022; Yang et al., 2022). Therefore, adequate clinical studies are required to confirm their clinical safety and efficacies.

## References

[B1] Altieri B. Grant W. B. Della Casa. S. Orio F. Pontecorvi A. Colao A. (2017). Vitamin D and pancreas: The role of sunshine vitamin in the pathogenesis of diabetes mellitus and pancreatic cancer. Crit. Rev. Food Sci. Nutr. 57 (16), 3472–3488. 10.1080/10408398.2015.1136922 27030935

[B2] Bu H. Q. Liu D. L. Wei W. T. Chen L. Huang H. Li Y. (2014). Oridonin induces apoptosis in SW1990 pancreatic cancer cells via p53-and caspase-dependent induction of p38 MAPK. Oncol. Rep. 31 (2), 975–982. 10.3892/or.2013.2888 24297112

[B3] Bu S. Z. Xu J. L. Sun J. H. (1999). Saikosaponin-d up-regulates GR mRNA expression and induces apoptosis in HL-60 cells. Chin. J. Hematol. 20 (7), 354–356. 11721431

[B4] Cai J. Shen W. Y. Zhang W. Y. Li G. Shen H. S. Li W. C. (2023). Xiao Chai Hu Tang alleviates the pancreatic tumorigenesis via improving the mtDNA N6-Methyladenine modification mediated mitochondrial dysfunction in Syrian hamster model. Phytomedicine 116, 154840. 10.1016/j.phymed.2023.154840 37172477

[B5] Cai Y. Y. Gao S. Ming L. (2017). The study of Scutellaria barbata inhibiting the proliferation, invasion, metastasis and tumorigenesis of PANC-1 cells by Hippo/YAP pathway in pancreatic cancer. J. Tradit. Chin. Med. 32, 2947–2951.

[B6] Cao L. Chen X. Xiao X. Ma Q. Y. Li W. (2016a). Resveratrol inhibits hyperglycemia-driven ROS-induced invasion and migration of pancreatic cancer cells via suppression of the ERK and p38 MAPK signaling pathways. Int. J. Oncol. 49 (2), 735–743. 10.3892/ijo.2016.3559 27278736

[B7] Cao L. Xiao X. Lei J. J. Duan W. X. Ma Q. Y. Li W. (2016b). Curcumin inhibits hypoxia-induced epithelial-mesenchymal transition in pancreatic cancer cells via suppression of the hedgehog signaling pathway. Oncol. Rep. 35 (6), 3728–3734. 10.3892/or.2016.4709 27035865

[B8] Chen H. Sun B. Pan S. Jian H. Sun X. (2009). Dihydroartemisinin inhibits growth of pancreatic cancer cells *in vitro* and *in vivo* . Anticancer Drugs 20 (2), 131–140. 10.1097/CAD.0b013e3283212ade 19209030

[B9] Chen L. Musa A. E. (2021). Boosting immune system against cancer by resveratrol. Phytother. Res. 35 (10), 5514–5526. 10.1002/ptr.7189 34101276

[B10] Chen P. Wang M. Wang C. (2019). Qingyihuaji formula reverses gemcitabine resistant human pancreatic cancer through regulate lncRNA AB209630/miR-373/EphB2-NANOG signals. Biosci. Rep. 39 (6), BSR20190610. 10.1042/BSR20190610 31147453 PMC6579980

[B11] Chen R. Y. Xu B. Chen S. F. Chen S. S. Zhang T. Ren J. (2014). Effect of oridonin-mediated hallmark changes on inflammatory pathways in human pancreatic cancer (BxPC-3) cells. World J. Gastroenterol. 20 (40), 14895–14903. 10.3748/wjg.v20.i40.14895 25356049 PMC4209552

[B12] Chen S. Li R. Chen Y. Chou C. K. Zhang Z. Yang Y. (2022). Scutellarin enhances anti-tumor immune responses by reducing TNFR2-expressing CD4+Foxp3+ regulatory T cells. Biomed. Pharmacother. 151, 113187. 10.1016/j.biopha.2022.113187 35676787

[B13] Chen Y. Guo Q. Q. Zhang B. Kang M. X. Xie Q. P. Wu Y. L. (2012). Bufalin enhances the antitumor effect of gemcitabine in pancreatic cancer. Oncol. Lett. 4 (4), 792–798. 10.3892/ol.2012.783 23205102 PMC3506674

[B14] Cho Y. R. Lee J. H. Kim J. H Kim S. J. Yoo H. J. Pack C. G. (2018). Matrine suppresses KRAS-driven pancreatic cancer growth by inhibiting autophagy-mediated energy metabolism. Mol. Oncol. 12 (7), 1203–1215. 10.1002/1878-0261.12324 29791786 PMC6026868

[B15] Chowdhury P. Jayroe J. J. White B. E. Fenton E. R. (2018). Effects of a natural polyphenol on nicotine-induced pancreatic cancer cell proliferation. Tob. Induc. Dis. 16, 50. 10.18332/tid/95159 31516447 PMC6659559

[B16] Dai H. Jiang Y. Luo Y. Bie P. Chen Z. Y. (2019). Triptolide enhances TRAIL sensitivity of pancreatic cancer cells by activating autophagy via downregulation of PUM1. Phytomedicine 62, 152953. 10.1016/j.phymed.2019.152953 31128486

[B17] Deng Y. Jia B. Y. Bian W. S. (2014). Research progress of treating pancreatic cancer with traditional Chinese medicine. Inf. Tradit. Chin. Med. 31, 170–172.

[B18] DhasmanaA.DhasmanaS.KotnalaS.LaskarP.KhanS.HaqueS. (2023). CEACAM7 expression contributes to early events of pancreatic cancer. J. Adv. Res. S2090-1232 (23), 00064–4. 10.1016/j.jare.2023.02.013 36828119

[B19] Ding X. Zhou X. Jiang B. Zhao Q. Zhou G. X. (2015). Triptolide suppresses proliferation, hypoxia-inducible factor-1α and c-Myc expression in pancreatic cancer cells. Mol. Med. Rep. 12 (3), 4508–4513. 10.3892/mmr.2015.3960 26094625

[B20] Du J. Wang X. Li Y. Ren X. Zhou Y. Hu W. (2021). DHA exhibits synergistic therapeutic efficacy with cisplatin to induce ferroptosis in pancreatic ductal adenocarcinoma via modulation of iron metabolism. Cell Death Dis. 12 (7), 705. 10.1038/s41419-021-03996-y 34262021 PMC8280115

[B21] Duan J. J. Yue W. Malhotra J. Y. Lu J. S. Gu E. J. Xu F. (2016). *In vitro* comparative studies of resveratrol and triacetylresveratrol on cell proliferation, apoptosis, and STAT3 and NFκB signaling in pancreatic cancer cells. Sci. Rep. 6, 31672. 10.1038/srep31672 27539371 PMC4990919

[B22] Fang S. Wang L. Luo C. Yi H. Wang X. Ning B. (2022). Curcumol inhibits the growth of xenografttumors in mice and the biological activities of pancreatic cancer cells by regulating the miR-21-5p/SMAD7 axis. Cell Cycle 21 (12), 1249–1266. 10.1080/15384101.2022.2046983 35253605 PMC9132406

[B23] Feng D. Z. Bai Y. Wen X. Y. (2010). Effects of PAMD on the expression of K-ras in pancreatic cancer tumor tissues. Inf. Tradit. Chin. Med. 27, 112–114.

[B24] Feng J. Rao M. Wan M. Liang L. Chen Z. Pang X. F. (2019). Triptolide suppresses pancreatic cancer cell proliferation by inhibiting hedgehog signaling pathway activity. Sci. China Life Sci. 62 (10), 1409–1412. 10.1007/s11427-018-9477-3 30900168

[B25] Fiala M. (2015). Curcumin and omega-3 fatty acids enhance NK cell-induced apoptosis of pancreatic cancer cells but curcumin inhibits interferon-γ production: Benefits of omega-3 with curcumin against cancer. Molecules 20 (2), 3020–3026. 10.3390/molecules20023020 25685909 PMC6272437

[B26] Florio R. DeFilippis B. Veschi S. di Giacomo V. Lanuti P. Catitti G. (2023). Resveratrol derivative exhibits marked antiproliferative actions, affecting stemness in pancreatic cancer cells. Int. J. Mol. Sci. 24 (3), 1977. 10.3390/ijms24031977 36768301 PMC9916441

[B27] Fu R. Yu F. Wu W. Liu J. Li J. Guo F. (2021). Bufalin enhances the killing efficacy of NK cells against hepatocellular carcinoma by inhibiting MICA shedding. Int. Immunopharmacol. 101, 108195. 10.1016/j.intimp.2021.108195 34678691

[B28] Glienke W. Maute L. Wicht J. Bergmann L. (2010). Curcumin inhibits constitutive STAT3 phosphorylation in human pancreatic cancer cell lines and downregulation of survivin/BIRC5 gene expression. Cancer Invest. 28 (2), 166–171. 10.3109/07357900903287006 20121547

[B29] Grau L. Soucek R. Pujol M. D. (2023). Resveratrol derivatives: Synthesis and their biological activities. Eur. J. Med. Chem. 246, 114962. 10.1016/j.ejmech.2022.114962 36463729

[B30] Gui Z. Li S. Liu X. Xu B. Xu J. (2015). Oridonin alters the expression profiles of microRNAs in BxPC-3 human pancreatic cancer cells. BMC Complement. Altern. Med. 15, 117. 10.1186/s12906-015-0640-5 25880988 PMC4399397

[B31] Guo H. C. Bu H. Q. Luo J. Wei W. T. Liu D. L. Chen H. (2012). Emodin potentiates the antitumor effects of gemcitabine in PANC-1 pancreatic cancer xenograft model *in vivo* via inhibition of inhibitors of apoptosis. *Int. J. Onc*ol. 40 (6), 1849–1857. 10.3892/ijo.2012.1389 22378302

[B32] Guo J. Chen T. Ma Z. Qiao C. Yuan F. Guo X. (2020). Oridonin inhibits 4T1 tumor growth by suppressing Treg differentiation via TGF-β receptor. Int. Immunopharmacol. 88, 106831. 10.1016/j.intimp.2020.106831 32853925

[B33] Guo Q. Chen Y. Zhang B. Kang M. X. Xie Q. P. Wu Y. L. (2009). Potentiation of the effect of gemcitabine by emodin in pancreatic cancer is associated with survivin inhibition. Biochem. Pharmacol. 77 (11), 1674–1683. 10.1016/j.bcp.2009.02.021 19428321

[B34] He J. Yin P. Xu K. (2020). Effect and molecular mechanisms of traditional Chinese medicine on tumor targeting tumor-associated macrophages. Drug Des. Devel Ther. 14, 907–919. 10.2147/DDDT.S223646 PMC7053810 32184560

[B35] Hoca M. Becer E. Kabaday H. Yücecan S. Vatansever H. S. (2020). The effect of resveratrol and quercetin on epithelial-mesenchymal transition in pancreatic cancer stem cell. Nutr. Cancer 72 (7), 1231–1242. 10.1080/01635581.2019.1670853 31595775

[B36] Hu H. Wang Z. Tan C. Liu X. Zhang H. Li K. (2020). Dihydroartemisinin/miR-29b combination therapy increases the pro-apoptotic effect of dihydroartemisinin on cholangiocarcinoma cell lines by regulating Mcl-1 expression. Adv. Clin. Exp. Med. 29 (8), 911–919. 10.17219/acem/121919 32790250

[B37] Huang M. Xin W. (2018). Matrine inhibiting pancreatic cells epithelial-mesenchymal transition and invasion through ROS/NF-κB/MMPs pathway. Life Sci. 192, 55–61. 10.1016/j.lfs.2017.11.024 29155301

[B38] Huang Q. Zhang Y. Zheng Y. Yang H. Yang Y. Mo Y. (2022). Molecular mechanism of curcumin and its analogs as multifunctional compounds against pancreatic cancer. Nutr. Cancer 74 (9), 3096–3108. 10.1080/01635581.2022.2071451 35583289

[B39] Jiang J. Liu R. Zhang Z. Zhang X. Qi R. Chen S. (2019). Study on the treatment of pancreatic cancer with integrated traditional Chinese and western medicine: A study protocol of a multicenter prospective cohort study. Med. Baltim. 98 (47), e17975. 10.1097/MD.0000000000017975 PMC6882619 31764804

[B40] Jiang Z. D. Chen X. Chen K. Sun L. K. Gao L. P. Zhou C. C. (2016). YAP inhibition by resveratrol via activation of AMPK enhances the sensitivity of pancreatic cancer cells to gemcitabine. Nutrients 8 (10), 546. 10.3390/nu8100546 27669292 PMC5083973

[B41] Klein A. P. (2021). Pancreatic cancer epidemiology: Understanding the role of lifestyle and inherited risk factors. Nat. Rev. Gastroenterol. Hepatol. 18 (7), 493–502. 10.1038/s41575-021-00457-x 34002083 PMC9265847

[B42] Kong R. Jia G. Cheng Z. X. Wang Y. W. Mu M. Wang S. J. (2012). Dihydroartemisinin enhances Apo2L/TRAIL-mediated apoptosis in pancreatic cancer cells via ROS-mediated up-regulation of death receptor 5. PLoS One 7 (5), e37222. 10.1371/journal.pone.0037222 22666346 PMC3364248

[B43] Kuo Y. T. Liao H. H. Chiang J. H. Wu M. Y. Chen B. C. Chang C. M. (2018). Complementary Chinese herbal medicine therapy improves survival of patients with pancreatic cancer in taiwan: A nationwide population-based cohort study. Integr. Cancer Ther. 17 (2), 411–422. 10.1177/1534735417722224 28774207 PMC6041895

[B44] Li B. Gan R. Yang Q. J. Huang J. L. Chen P. G. Guo C. (2015). Chinese herbal medicines as an adjunctive therapy for unresectable pancreatic cancer: A systematic review and meta-analysis. Evid. Based Complement. Altern. Med. 2015, 350730. 10.1155/2015/350730 PMC4670883 26681966

[B45] Li M. Wang M. M. Guo X. W. Wu C. Y. Li D. R. Zhang X. (2018). Different survival benefits of Chinese medicine for pancreatic cancer: How to choose? Chin. J. Integr. Med. 24 (3), 178–184. 10.1007/s11655-017-2971-1 29063468

[B46] Li M. Y. Yu X. J. Guo H. Sun L. M. Wang A. J. Liu Q. J. (2014). Bufalin exerts antitumor effects by inducing cell cycle arrest and triggering apoptosis in pancreatic cancer cells. Tumour Biol. 35 (3), 2461–2471. 10.1007/s13277-013-1326-6 24218335

[B47] Li N. Wang C. Zhang P. You S. Y. (2018). Emodin inhibits pancreatic cancer EMT and invasion by up-regulating microRNA-1271. Mol. Med. Rep. 18 (3), 3366–3374. 10.3892/mmr.2018.9304 30066876 PMC6102704

[B48] Li R. T. Fan Q. L. (2016). Research progress of treating pancreatic cancer with traditional Chinese medicine. Hunan J. Tradit. Chin. Med. 32, 183–185.

[B49] Li W. Cao L. Chen X. Lei J. J. Ma Q. Y. (2016). Resveratrol inhibits hypoxia-driven ROS-induced invasive and migratory ability of pancreatic cancer cells via suppression of the Hedgehog signaling pathway. Oncol. Rep. 35 (3), 1718–1726. 10.3892/or.2015.4504 26707376

[B50] Li W. Jiang Z. Xiao X. Wang Z. Wu Z. Ma Q. Y. (2018). Curcumin inhibits superoxide dismutase-induced epithelial-to-mesenchymal transition via the PI3K/Akt/NF-κB pathway in pancreatic cancer cells. Int. J. Oncol. 52 (5), 1593–1602. 10.3892/ijo.2018.4295 29512729

[B51] Li W. Ma Q. Li B. Han L. Liu J. B. Xu Q. (2013). Resveratrol inhibits the epithelial-mesenchymal transition of pancreatic cancer cells via suppression of the PI-3K/Akt/NF-κB pathway. Curr. Med. Chem. 20 (33), 4185–4194. 10.2174/09298673113209990251 23992306 PMC4085327

[B52] Li W. Sun L. Lei J. Wu Z. Ma Q. Wang Z. (2020). Curcumin inhibits pancreatic cancer cell invasion and EMT by interfering with tumor-stromal crosstalk under hypoxic conditions via the IL-6/ERK/NF-κB axis. Oncol. Rep. 44 (1), 382–392. 10.3892/or.2020.7600 32377752

[B53] Li W. Wang Z. Xiao X. Han L. Wu Z. Ma Q. Y. (2019). Curcumin attenuates hyperglycemia-driven EGF-induced invasive and migratory abilities of pancreatic cancer via suppression of the ERK and AKT pathways. Oncol. Rep. 41 (1), 650–658. 10.3892/or.2018.6833 30542713

[B54] Li Y. L. Wang Y. W. Kon R. Xu D. B. Pan S. H. Chen H. (2016). Dihydroartemisinin suppresses pancreatic cancer cells via a microRNA-mRNA regulatory network. Oncotarget 7 (38), 62460–62473. 10.18632/oncotarget.11517 27613829 PMC5308739

[B55] Li Y. Shen Y. Yao C. L. Guo D. A. (2020). Quality assessment of herbal medicines based on chemical fingerprints combined with chemometrics approach: A review. J. Pharm. Biomed. Anal. 185, 113215. 10.1016/j.jpba.2020.113215 32199327

[B56] Liu D. L. Bu H. Li H. Chen H. Guo H. C. Wang Z. H. (2012). Emodin reverses gemcitabine resistance in pancreatic cancer cells via the mitochondrial apoptosis pathway *in vitro* . Int. J. Oncol. 40 (4), 1049–1057. 10.3892/ijo.2011.1285 22159556 PMC3584653

[B57] Liu D. L. Bu H. Q. Jin H. M. Zhao J. F. Li Y. Huang H. (2014). Enhancement of the effects of gemcitabine against pancreatic cancer by oridonin via the mitochondrial caspase-dependent signaling pathway. Mol. Med. Rep. 10 (6), 3027–3034. 10.3892/mmr.2014.2584 25242370

[B58] Liu D. L. Bu H. Q. Wang W. L. Luo H. Cheng B. N. (2020). Oridonin enhances the anti-tumor activity of gemcitabine towards pancreatic cancer by stimulating Bax- and Smac-dependent apoptosis. Transl. Cancer Res. 9 (7), 4148–4161. 10.21037/tcr-19-3000 35117784 PMC8798810

[B59] Liu L. Salnikov A. V. Bauer N. Aleksandrowicz E. Labsch S. Nwaeburu C. (2014). Triptolide reverses hypoxia-induced epithelial-mesenchymal transition and stem-like features in pancreatic cancer by NF-κB downregulation. Int. J. Cancer 134 (10), 2489–2503. 10.1002/ijc.28583 24615157 PMC4255690

[B60] Liu Q. Q. Chen K. Ye Q. Jiang X. H. Sun Y. W. (2016). Oridonin inhibits pancreatic cancer cell migration and epithelial-mesenchymal transition by suppressing Wnt/β-catenin signaling pathway. Cancer Cell Int. 16, 57. 10.1186/s12935-016-0336-z 27453691 PMC4957915

[B61] Liu T. Y. Song Y. Chen H. Pan S. H. Sun X. Y. (2010). Matrine inhibits proliferation and induces apoptosis of pancreatic cancer cells *in vitro* and *in vivo* . Biol. Pharm. Bull. 33 (10), 1740–1745. 10.1248/bpb.33.1740 20930385

[B62] Liu X. Xiao X. Y. Shou Q. Y. Yan J. F. Chen L. Fu H. Y. (2016). Bufalin inhibits pancreatic cancer by inducing cell cycle arrest via the c-Myc/NF-κB pathway. J. Ethnopharmacol. 193, 538–545. 10.1016/j.jep.2016.09.047 27686271

[B63] Liu X. Zhou Y. Peng J. Xie B. Shou Q. Wang J. (2020). Silencing c-myc enhances the antitumor activity of bufalin by suppressing the HIF-1α/SDF-1/CXCR4 pathway in pancreatic cancer cells. Front. Pharmacol. 11, 495. 10.3389/fphar.2020.00495 32362830 PMC7181899

[B64] Liu Y. B. Su H. Su Y. M. (2015). Study on the regulation of the expression of K-ras and DPC4 protein by rhizome menispermi. Inf. Tradit. Chin. Med. 32, 10–12.

[B65] Lou S. Xu J. Wang B. Li S. Ren J. Hu Z. (2019). Downregulation of lncRNA AFAP1-AS1 by oridonin inhibits the epithelial-to-mesenchymal transition and proliferation of pancreatic cancer cells. Acta Biochim. Biophys. Sin. (Shanghai) 51 (8), 814–825. 10.1093/abbs/gmz071 31314060

[B66] Lv Y. H. Wan D. H. Kang Y. H. Huangpu C. S. Hu G. Q. Liu B. (2012). Cinnamaldehyde ofloxacin-3-ylhydrazone induces apoptosis of human pancreatic carcinoma cells. Chin. Pharmacol. J. 47 (14), 1119–1123.

[B67] Ma J. G. Xue M. W. Zhang S. M. Cheng L. Qian W. K. Duan W. X. (2019). Resveratrol inhibits the growth of tumor cells under chronic stress via the ADRB‑2‑HIF‑1α axis. Oncol. Rep. 41 (2), 1051–1058. 10.3892/or.2018.6894 30535465

[B68] Ma J. X. Sun Y. L. Yu Y. Zhan J. Wu H. Y. Yu X. F. (2019). Triptolide enhances the sensitivity of pancreatic cancer PANC-1 cells to gemcitabine by inhibiting TLR4/NF-κB signaling. Am. J. Transl. Res. 11 (6), 3750–3760. 31312385 PMC6614654

[B69] Ma J. Y. Li K. L. Shi S. L. Li J. Tang S. N. Liu L. H. (2022). The application of UHPLC-HRMS for quality control of traditional Chinese medicine. Front. Pharmacol. 13, 922488. 10.3389/fphar.2022.922488 35721122 PMC9201421

[B70] Ma Y. C. Zou F. Z. Xiong J. P. Wan W. Yin L. Li X. J. (2015). Effect of matrine on HPAC cell migration by down-regulating the expression of MT1-MMP via Wnt signaling. Cancer Cell Int. 15, 59. 10.1186/s12935-015-0210-4 26113801 PMC4480578

[B71] MacKenzie T. N. Mujumdar N. Banerjee S. Sangwan V. Sarver A. Vickers S. (2013). Triptolide induces the expression of miR-142-3p: A negative regulator of heat shock protein 70 and pancreatic cancer cell proliferation. Mol. Cancer Ther. 12 (7), 1266–1275. 10.1158/1535-7163.MCT-12-1231 23635652 PMC3707985

[B72] Maisonneuve P. (2019). Epidemiology and burden of pancreatic cancer. Presse Med. 48 (3), e113–e123. 10.1016/j.lpm.2019.02.030 30878335

[B73] Malhotra L. Sharma S. Hariprasad G. Dhingra R. Mishra V. Sharma R. S. (2022). Mechanism of apoptosis activation by Curcumin rescued mutant p53Y220C in human pancreatic cancer. Biochim. Biophys. Acta Mol. Cell Res. 1869 (12), 119343. 10.1016/j.bbamcr.2022.119343 36007676

[B74] McGuigan A. Kelly P. Turkington R. C. Jones C. Coleman H. G. McCain R. S. (2018). Pancreatic cancer: A review of clinical diagnosis, epidemiology, treatment and outcomes. World J. Gastroenterol. 24 (43), 4846–4861. 10.3748/wjg.v24.i43.4846 30487695 PMC6250924

[B75] Meng D. Bai Y. Su Y. M. (2010). Effects of PAMD on tumor inhibition of BXPC-3 in tumor-bearing mice and TNF-α content in peripheral blood. Inf. Tradit. Chin. Med. 27, 29–31.

[B76] Miller A. L. Garcia P. L. Yoon K. J. (2020). Developing effective combination therapy for pancreatic cancer: An overview. Pharmacol. Res. 155, 104740. 10.1016/j.phrs.2020.104740 32135247 PMC7365261

[B77] Mu L. Wu P. Zhang Y. Li S. Yang R. Wang S. (2022). Development of a novel oral complex lipid emulsion containing triptolide for targeting pancreatic cancer. Pharm. Dev. Technol. 27 (8), 881–891. 10.1080/10837450.2022.2127767 36154850

[B78] Nagaraju G. P. Benton L. Bethi S. R. Shoji M. El-Rayes B. F. (2019). Curcumin analogs: Their roles in pancreatic cancer growth and metastasis. Int. J. Cancer 145 (1), 10–19. 10.1002/ijc.31867 30226272

[B79] Nagaraju G. P. Zhu S. Ko J. E. AshrithaKandimallaSnyde N. R. J. P. Shoji M. El-Rayes B. F. (2015). Antiangiogenic effects of a novel synthetic curcumin analogue in pancreatic cancer. *Cancer Lett*. 357 (2), 557–565. 10.1016/j.canlet.2014.12.007 25497868

[B80] Ni Y. Q. You J. L. Gong S. X. (2013b). Clinical study on the effect of WD-3 on survival of patients with advanced pancreatic cancer. Liaoning J. Tradit. Chin. Med. 40, 84–86.

[B81] Ni Y. Q. You J. L. Gong S. X. (2013a). Clinical study on the effect of WD-3 or combined chemotherapy on the quality of life of patients with advanced pancreatic cancer. Jiangsu J. Tradit. Chin. Med. 45, 29–31.

[B82] Ni Y. Q. You J. L. Yang Z. X. (2006). WD-3 combined with western medicine in treating 21 cases of advanced pancreatic cancer. Shaanxi J. Tradit. Chin. Med. 2006, 426–427.

[B83] Ning X. Y. Du Y. Q. Ben Q. W. Huang L. He X. P. Gong Y. F. (2016). Bulk pancreatic cancer cells can convert into cancer stem cells (CSCs) *in vitro* and 2 compounds can target these CSCs. Cell Cycle 15 (3), 403–412. 10.1080/15384101.2015.1127471 26709750 PMC4943690

[B84] Ning Z. Y. Zhu Z. F. Wang H. Y. Zhang C. Y. Xu L. T. Zhuang L. P. (2019). High-intensity focused ultrasound enhances the effect of bufalin by inducing apoptosis in pancreatic cancer cells. Onco Targets Ther. 12, 1161–1170. 10.2147/OTT.S185953 30863083 PMC6388946

[B85] OuyangLiu H. Q. L. M. Chen Z. Luo J. M. Yu E. X. (2010). Effects of Qingyi Huaji decoction on serum levels of interleukin-6, interleukin-8 and tumor necrosis factor-alpha in nude mice bearing pancreatic tumors. Chin. J. Integr. Med. 8 (7), 655–661. 10.3736/jcim20100709 20619142

[B86] Pan F. P. Zhou H. K. Bu H. Q. Chen Z. Q. Zhang H. Xu L. P. (2016). Emodin enhances the demethylation by 5-Aza-CdR of pancreatic cancer cell tumor-suppressor genes P16, RASSF1A and ppENK. Oncol. Rep. 35 (4), 1941–1949. 10.3892/or.2016.4554 26782786 PMC4774670

[B87] Peng X. C. Huang J. R. Wang S. W. Liu L. Liu Z. Z. Sethi G. (2018). Traditional Chinese Medicine in neuroprotection after brain insults with special reference to radioprotection. Evid. Based Complement. Altern. Med. 2018, 2767208. 10.1155/2018/2767208 PMC6287144 30598683

[B88] Periyasamy L. Muruganantham B. Park W. Y. Muthusami S. (2022). Phyto-targeting the CEMIP expression as a strategy to prevent pancreatic cancer metastasis. Curr. Pharm. Des. 28 (11), 922–946. 10.2174/1381612828666220302153201 35236267

[B89] Phillips P. A. Dudej V. McCarroll J. A. Borja-CachoDawraGrizzle D. R. K. W. E. Vickers S. M. Saluja A. K. (2007). Triptolide induces pancreatic cancer cell death via inhibition of heat shock protein. Cancer Res. 67 (19), 9407–9416. 10.1158/0008-5472.CAN-07-1077 17909050

[B90] Qian X. Chen Z. Chen S. S. Liu L. M. Zhang A. Q. (2020). Integrated analyses identify immune-related signature associated with qingyihuaji formula for treatment of pancreatic ductal adenocarcinoma using network pharmacology and weighted gene Co-expression network. J. Immunol. Res. 2020, 7503605. 10.1155/2020/7503605 32537471 PMC7256764

[B91] Qiu W. Chen R. Chen X. Zhang H. Song L. Cui W. (2018). Oridonin-loaded and GPC1-targeted gold nanoparticles for multimodal imaging and therapy in pancreatic cancer. Int. J. Nanomedicine 13, 6809–6827. 10.2147/IJN.S177993 30425490 PMC6205542

[B92] Qiu X. N. Cui L. H. Zhang S. K. (2016). Progress in the experimental study on the prevention and treatment of pancreatic cancer with Chinese herbal monomers and their effective components. Chin. J. Integr. Tradit. West Med. 22, 412–414.

[B93] Schizas D. Charalampakis N. Kole C. Economopoulou P. Koustas E. Gkotsis E. (2020). Immunotherapy for pancreatic cancer: A 2020 update. Cancer Treat. Rev. 86, 102016. 10.1016/j.ctrv.2020.102016 32247999

[B94] Sellam F. Harir N. Khale M. B. Mrabent N. M. Salah R. Diaf M. (2015). Epidemiology and risk factors for exocrine pancreatic cancer in a Northern African population. J. *Gastrointest. Cancer* 46 (2), 126–130. 10.1007/s12029-015-9693-4 25737417

[B95] Shen Y. H. Liu L. M. (2009). Survival analysis on 64 cases of advanced pancreatic cancer treated by integrated Western and Traditional Chinese Medicine mainly with Qingyi Huaji Formula. J. Tradit. Chin. Med. 50, 39–42.

[B96] Song L. B. Gao S. Zhang A. Q. Qian X. Liu L. M. (2017). Babaodan Capsule combined with Qingyi Huaji Formula in advanced pancreatic cancer-A feasibility study. Chin. J. Integr. Med. 23 (12), 937–942. 10.1007/s11655-017-2279-1 28664246

[B97] Song L. B. Liu L. M. Chen H. (2018). Retrospective study of 232 post-operative patients with pancreatic cancer treated by modified Qingyi Huaji formula combined with western medicine. Chin. J. Integr. Med. 38, 932–935.

[B98] Su Y. M. Zhang C. Xiao J. Y. (2007). Effects of PAMD on the proliferation of human tumour cells of PC-3 and BT5637. J. Harbin Med. Univ. 2007, 129–131.

[B99] Subramaniam D. Kaushik G. Dandawate P. Anant S. (2018). Targeting cancer stem cells for chemoprevention of pancreatic cancer. Curr. Med. Chem. 25 (22), 2585–2594. 10.2174/0929867324666170127095832 28137215 PMC5630517

[B100] Tong H. Huang Z. Chen H. Zhou B. Liao Y. Wang Z. (2020). Emodin reverses gemcitabine resistance of pancreatic cancer cell lines through inhibition of IKKβ/NF-κB signaling pathway. Onco Targets Ther. 13, 9839–9848. 10.2147/OTT.S253691 33061461 PMC7537840

[B101] Wan Y. Xu L. Liu Z. Yang M. Jiang X. Zhang Q. (2019). Utilising network pharmacology to explore the underlying mechanism of Wumei Pill in treating pancreatic neoplasms. BMC Complement. Altern. Med. 19 (1), 158. 10.1186/s12906-019-2580-y 31272505 PMC6611005

[B102] Wang G. Guo H. Ren Y. Chen W. Wang Y. Li J. (2023). Triptolide enhances carboplatin-induced apoptosis by inhibiting nucleotide excision repair (NER) activity in melanoma. Front. Pharmacol. 14, 1157433. 10.3389/fphar.2023.1157433 37324464 PMC10267402

[B103] Wang H. Wu Z. Fan X. Wu C. Lu S. Geng L. (2023). Identification of key pharmacological components and targets for Aidi injection in the treatment of pancreatic cancer by UPLC-MS, network pharmacology, and *in vivo* experiments. Chin. Med. 18 (1), 7. 10.1186/s13020-023-00710-2 36641437 PMC9840244

[B104] Wang H. Y. Ning Z. Y. Li Y. Y. Zhu X. Y. Meng Z. Q. (2016). Bufalin suppresses cancer stem-like cells in gemcitabine-resistant pancreatic cancer cells via Hedgehog signaling. Mol. Med. Rep. 14 (3), 1907–1914. 10.3892/mmr.2016.5471 27432228 PMC4991682

[B105] Wang J. M. Bai Y. Wen X. Y. (2010). Effect of PAMD on the expression of DPC4 protein in pancreatic cancer tumor. Acta Chin. Med. Pharm. 38, 27–29.

[B106] Wang J. Wang Q. Zhang P. Zhang R. He J. (2022). Efficacy and safety of traditional Chinese medicine for the treatment of pancreatic cancer: An overview of systematic reviews and meta-analyses. Front. Pharmacol. 13, 896017. 10.3389/fphar.2022.896017 36120323 PMC9475193

[B107] Wang L. J. Lu J. Z. (2016). Research progress of treating pancreatic cancer with traditional Chinese medicine. J. Tradit. Chin. Med. 31, 961–964.

[B108] Wang L. Xu J. Yan Y. Liu H. Karunakaran T. Li F. (2019). Green synthesis of gold nanoparticles from Scutellaria barbata and its anticancer activity in pancreatic cancer cell (PANC-1). Artif. Cells Nanomed Biotechnol. 47 (1), 1617–1627. 10.1080/21691401.2019.1594862 31014134

[B109] Wang P. Liu L. M. Chen Z. (2010). Effect of Qingyi Huaji formula for inhibition of pancreatic cancer cell growth through down-regulating Ski expression. Chin. J. Integr. Tradit. West Med. 30 (9), 942–945. 21179734

[B110] Wang Q. Qu C. Xie F. Chen L. Y. Liu L. M. Liang X. H. (2017). Curcumin suppresses epithelial-to-mesenchymal transition and metastasis of pancreatic cancer cells by inhibiting cancer-associated fibroblasts. Am. J. Cancer Res. 7 (1), 125–133. 28123853 PMC5250686

[B111] Wang S. J. Gao Y. Chen H. Kon R. Jiang H. C. Pan S. H. (2010). Dihydroartemisinin inactivates NF-kappaB and potentiates the anti-tumor effect of gemcitabine on pancreatic cancer both *in vitro* and *in vivo* . Cancer Lett. 293 (1), 99–108. 10.1016/j.canlet.2010.01.001 20137856

[B112] Wang S. J. Sun B. Cheng Z. X. Zhou H. X. Gao Y. Kon R. (2011). Dihydroartemisinin inhibits angiogenesis in pancreatic cancer by targeting the NF-κB pathway. Cancer Chemother. Pharmacol. 68 (6), 1421–1430. 10.1007/s00280-011-1643-7 21479633

[B113] Wang S. Long S. Wu W. (2018). Application of traditional Chinese medicines as personalized therapy in human cancers. Am. J. Chin. Med. 46 (5), 953–970. 10.1142/S0192415X18500507 29986595

[B114] Wang Y. Chen F. Zhou H. Huang L. Ye J. Liu X. (2023). Redox dyshomeostasis with dual stimuli-activatable dihydroartemisinin nanoparticles to potentiate ferroptotic therapy of pancreatic cancer. Small Methods 7 (5), e2200888. 10.1002/smtd.202200888 36446643

[B115] Wei D. M. Chen H. Yin G. (2015). Study on the mechanism of PAMD to regulate Gli1 gene against pancreatic cancer. Yunnan J. Tradit. Chin. Med. Mater medica 36, 64–65.

[B116] Wei W. T. Wang J. F. Hu Y. Q. Chen S. Liu J. S. (2022). Emodin reverses resistance to gemcitabine in pancreatic cancer by suppressing stemness through regulation of the epithelial-mesenchymal transition. Exp. Ther. Med. 225 (1), 7. 10.3892/etm.2022.11706 PMC9748633 36545274

[B117] Wong W. Chen B. Z. Lee A. K. Y. Chan A. H. C. Wu J. C. Y. Lin Z. (2019). Chinese herbal medicine effectively prolongs the overall survival of pancreatic cancer patients: A case series. Integr. Cancer Ther. 18, 1534735419828836. 10.1177/1534735419828836 30791742 PMC6432679

[B118] Wu C. X. Bai Y. Sun H. Y. (2019). PAMD inhibits the proliferation of pancreatic cancer BXPC-3 cells and its effect on the Hh signaling pathway. *J. Liaoning Univ. Tradit. Chin. Med*. 21, 59–62.

[B119] Xie C. Liang C. Wang R. Yi K. Zhou X. Li X. (2023). Resveratrol suppresses lung cancer by targeting cancer stem-like cells and regulating tumor microenvironment. J. Nutr. Biochem. 112, 109211. 10.1016/j.jnutbio.2022.109211 36370924

[B120] Xu B. Shen W. Liu X. Zhang T. Ren J. Fan Y. J. (2015). Oridonin inhibits BxPC-3 cell growth through cell apoptosis. *Acta Biochim. Biophys. Sin*. (Shanghai). 47 (3), 164–173. 10.1093/abbs/gmu134 25651847

[B121] Xu P. L. Cheng C. S. Jiao J. Y. Chen H. Chen Z. Li P. (2022). Matrine injection inhibits pancreatic cancer growth via modulating carbonic anhydrases-a network pharmacology-based study with *in vitro* validation. J. Ethnopharmacol. 287, 114691. 10.1016/j.jep.2021.114691 34597654

[B122] Xu Y. L. Zhu F. Y. Xu S. Liu L. M. (2015). Anti-tumor effect of the extract from qingyihuaji formula on pancreatic cancer by down-regulating Notch-4 and Jagged-1. J. Tradit. Chin. Med. 35 (1), 77–83. 10.1016/s0254-6272(15)30012-1 25842732

[B123] Xue Q. You J. L. Gong S. X. (2016). Clinical analysis of Chinese medicine WD-3 combined with Xiaozhenggao to treat advanced pancreatic cancer. J. Liaoning Univ. Tradit. Chin. Med. 18, 184–186.

[B124] Yan C. LuoLi Z. Wen. Li X. Dallmann R. Kurihara H. Li Y. F. (2020). Disturbed yin-yang balance: Stress increases the susceptibility to primary and recurrent infections of herpes simplex virus type 1. Acta Pharm. Sin. B 10 (3), 383–398. 10.1016/j.apsb.2019.06.005 32140387 PMC7049575

[B125] Yang H. Z. Yang T. Gong D. D. Li X. H. Su G. X. Guo P. (2022). A trinity fingerprint evaluation system of traditional Chinese medicine. J. Chromatogr. A 1673, 463118. 10.1016/j.chroma.2022.463118 35550981

[B126] Yang S. W. Wang W. Xie X. Y. Zhu W. P. Li F. Q. (2011). *In vitro* synergistic cytotoxic effect of triptolide combined with hydroxycamptothecin on pancreatic cancer cells. Am. J. Chin. Med. 39 (1), 121–134. 10.1142/S0192415X11008695 21213403

[B127] Yang X. Hao J. Zhu C. H. Niu Y. Y. Ding X. L. Liu C. (2015). Survival benefits of western and traditional Chinese medicine treatment for patients with pancreatic cancer. Med. Baltim. 94 (26), e1008. 10.1097/MD.0000000000001008 PMC4504629 26131801

[B128] Yao W. T. Maitra A. Ying H. Q. (2020). Recent insights into the biology of pancreatic cancer. EBioMedicine 53, 102655. 10.1016/j.ebiom.2020.102655 32139179 PMC7118569

[B129] Yao W. Y. Zhou Y. F. Qia A. H. Zhang Y. P. Qiao M. M. Zhai Z. K. (2015). Emodin has a protective effect in cases of severe acute pancreatitis via inhibition of nuclear factor-κB activation resulting in antioxidation. Mol. Med. Rep. 11 (2), 1416–1420. 10.3892/mmr.2014.2789 25351888

[B130] Yu Z. Li Y. Li Y. Zhang J. Li M. Ji L. (2022). Bufalin stimulates antitumor immune response by driving tumor-infiltrating macrophage toward M1 phenotype in hepatocellular carcinoma. J. Immunother. Cancer 10 (5), e004297. 10.1136/jitc-2021-004297 35618286 PMC9125767

[B131] Zhang B. Yuan Q. Zhang B. Li S. Wang Z. Liu H. (2023). Characterization of neuroendocrine regulation- and metabolism-associated molecular features and prognostic indicators with aid to clinical chemotherapy and immunotherapy of patients with pancreatic cancer. Front. Endocrinol. (Lausanne) 13, 1078424. 10.3389/fendo.2022.1078424 36743929 PMC9895410

[B132] Zhang C. HeLi X. J,L. Lu C. Lu A. P. (2017). Effect of the natural product triptolide on pancreatic cancer: A systematic review of preclinical studies. Front. Pharmacol. 8, 490. 10.3389/fphar.2017.00490 28890697 PMC5574901

[B133] Zhang H. B. Zhuo Y. Z. Li D. H. Zhang L. Q. Gao Q. Y. Yang L. (2022). Dihydroartemisinin inhibits the growth of pancreatic cells by inducing ferroptosis and activating antitumor immunity. Eur. J. Pharmacol. 926, 175028. 10.1016/j.ejphar.2022.175028 35569552

[B134] Zhang H. Q. Li S. J. Qiao Q. Z. (2010). A randomized controlled study of QYHJ formula in the treatment of advanced pancreatic cancer. Chin. Remedies Clin. 10, 1415–1416.

[B135] Zhang J. J. Chen Z. Shi W. D. (2008). Effect of Qingyi Huaji formula on serum immunosuppressive factors and immune function of splenic lymphocytes in mice with pancreatic cancer. Chin. J. Exp. Tradit. Med. Form. 2008, 49–51.

[B136] Zhang L. Zhou J. Yan Y. Zhou X. Zhou Q. Du R. (2019). Excipient-free nanodispersion of 7-ethyl-10-hydroxycamptothecin exerts potent therapeutic effects against pancreatic cancer cell lines and patient-derived xenografts. Cancer Lett. 465, 36–44. 10.1016/j.canlet.2019.08.019 31479691

[B137] Zhang M. W. He Y. Wei M. X. (2023). Design, synthesis and biological evaluation of matrine-dithiocarbamate hybrids as potential anticancer agents. Eur. J. Med. Chem. 254, 115375. 10.1016/j.ejmech.2023.115375 37084600

[B138] Zhang Q. Chen W. W. Sun X. Qian D. Tang D. D. Zhang L. L. (2022). The versatile emodin: A natural easily acquired anthraquinone possesses promising anticancer properties against a variety of cancers. Int. J. Biol. Sci. 18 (8), 3498–3527. 10.7150/ijbs.70447 35637953 PMC9134920

[B139] Zhang W. Chen H. Liu D. L. Li H. Luo J. Zhang J. H. (2013). Emodin sensitizes the gemcitabine-resistant cell line Bxpc-3/Gem to gemcitabine via downregulation of NF-κB and its regulated targets. Int. J. Oncol. 42 (4), 1189–1196. 10.3892/ijo.2013.1839 23440366

[B140] Zhang X. Liu Q. Liao Q. Zhao Y. (2020). Pancreatic cancer, gut microbiota, and therapeutic efficacy. J. Cancer 11 (10), 2749–2758. 10.7150/jca.37445 32226493 PMC7086274

[B141] Zhao X. Zhang Q. Wang Y. Li S. Yu X. Wang B. (2021). Oridonin induces autophagy-mediated cell death in pancreatic cancer by activating the c-Jun N-terminal kinase pathway and inhibiting phosphoinositide 3-kinase signaling. Ann. Transl. Med. 9 (13), 1084. 10.21037/atm-21-2630 34422996 PMC8339817

[B142] Zhong L. L. Bai Y. Fei H. X. (2014). Research of PAMD and pancreatic cancer. J. Liaoning Univ. Tradit. Chin. Med. 16, 85–88.

[B143] Zhong Z. X. Li X. Z. Liu J. T. Qin N. Duan H. Q. Duan X. C. (2023). Disulfide bond-based SN38 prodrug nanoassemblies with high drug loading and reduction-triggered drug release for pancreatic cancer therapy. Int. J. Nanomedicine 18, 1281–1298. 10.2147/IJN.S404848 36945256 PMC10024910

[B144] Zhou H. B. Chen J. M. Sha o. L. M. Chen Z. G. (2015). Apoptosis of human pancreatic carcinoma cell-1 cells induced by Yin Chen Hao Decoction. World J. Gastroenterol. 21 (27), 8352–8357. 10.3748/wjg.v21.i27.8352 26217086 PMC4507104

[B145] Zhou Q. Xiang H. Liu H. Qi B. Shi X. Guo W. (2021). Emodin alleviates intestinal barrier dysfunction by inhibiting apoptosis and regulating the immune response in severe acute pancreatitis. Pancreas 50 (8), 1202–1211. 10.1097/MPA.0000000000001894 34714285 PMC8565508

[B146] Zhou Z. G. Zhang C. Y. Fei H. X. Zhong L. L. Bai Y. (2015). Phenolic alkaloids from Menispermum dauricum inhibits BxPC-3 pancreatic cancer cells by blocking of Hedgehog signaling pathway. Pharmacogn. Mag. 11 (44), 690–697. 10.4103/0973-1296.165548 26600712 PMC4621636

[B147] Zhu M. M. Zhang Q. Wang X. L. Kang L. C. Yang Y. N. Liu Y. S. (2016). Metformin potentiates anti-tumor effect of resveratrol on pancreatic cancer by down-regulation of VEGF-B signaling pathway. Oncotarget 7 (51), 84190–84200. 10.18632/oncotarget.12391 27705937 PMC5356654

[B148] Zhu Y. Bu S. (2017). Curcumin induces autophagy, apoptosis, and cell cycle arrest in human pancreatic cancer cells. Evid. Based Complement. Altern. Med. 2017, 5787218. 10.1155/2017/5787218 PMC5610853 29081818

[B149] Zhuang H. W. Dai X. D. Zhang X. Y. Mao Z. Q. Huang H. J. (2020). Sophoridine suppresses macrophage-mediated immunosuppression through TLR4/IRF3 pathway and subsequently upregulates CD8+ T cytotoxic function against gastric cancer. Biomed. Pharmacother. 121, 109636. 10.1016/j.biopha.2019.109636 31733580

